# The Influence of Chromosomal Environment on X-Linked Gene Expression in *Drosophila melanogaster*

**DOI:** 10.1093/gbe/evaa227

**Published:** 2020-10-26

**Authors:** Aleksei Belyi, Eliza Argyridou, John Parsch

**Affiliations:** Division of Evolutionary Biology, Faculty of Biology, LMU Munich, Planegg-Martinsried, Germany

**Keywords:** sex chromosomes, dosage compensation, gene regulation, sex-biased expression, reporter gene

## Abstract

Sex chromosomes often differ from autosomes with respect to their gene expression and regulation. In *Drosophila melanogaster*, X-linked genes are dosage compensated by having their expression upregulated in the male soma, a process mediated by the X-chromosome-specific binding of the dosage compensation complex (DCC). Previous studies of X-linked gene expression found a negative correlation between a gene’s male-to-female expression ratio and its distance to the nearest DCC binding site in somatic tissues, including head and brain, which suggests that dosage compensation influences sex-biased gene expression. A limitation of the previous studies, however, was that they focused on endogenous X-linked genes and, thus, could not disentangle the effects of chromosomal position from those of gene-specific regulation. To overcome this limitation, we examined the expression of an exogenous reporter gene inserted at many locations spanning the X chromosome. We observed a negative correlation between the male-to-female expression ratio of the reporter gene and its distance to the nearest DCC binding site in somatic tissues, but not in gonads. A reporter gene’s location relative to a DCC binding site had greater influence on its expression than the local regulatory elements of neighboring endogenous genes, suggesting that intra-chromosomal variation in the strength of dosage compensation is a major determinant of sex-biased gene expression. Average levels of sex-biased expression did not differ between head and brain, but there was greater positional effect variation in the brain, which may explain the observed excess of endogenous sex-biased genes located on the X chromosome in this tissue.

Significance

In many species, sex chromosomes differ in their copy number and gene regulation between males and females. Here, we show that in *Drosophila melanogaster* a gene’s location on the X chromosome influences its expression ratio between the sexes and that this effect is associated with the mechanism of sex chromosome dosage compensation. Our results help explain previously observed differences in X-linked gene expression between the sexes and among tissues.

## Introduction

In species that reproduce sexually, females and males typically differ in their morphology, behavior, gene expression, and chromosomal content. In *Drosophila melanogaster*, sex is determined by a pair of heteromorphic sex chromosomes, with females being homogametic (XX) and males heterogametic (XY) (Salz and Erickson 2010). The X chromosome contains over 2,000 genes, which is proportionate to its size (about 16% of the genome), whereas the Y chromosome is almost completely heterochromatic and contains only 12 protein-coding genes (Vibranovski et al. 2008; Thurmond et al. 2019). As a result of its monosomy in males, the X chromosome has evolved a number of differences from the autosomes in its gene content and regulation (Meisel and Connallon 2013; Huylmans and Parsch 2015; Grath and Parsch 2016; Kuroda et al. 2016; Landeen et al. 2016).

A major regulatory difference between the X chromosome and the autosomes is the sex chromosome dosage compensation that occurs in the male soma, where the transcription of genes on the single male X chromosome is increased ∼2-fold to balance the expression of the X chromosome and autosomes in males, and of the single X chromosome in males with the two copies in females (Ercan 2015). Somatic dosage compensation is mediated by the X-chromosome-specific binding of the dosage compensation complex (DCC), which is composed of the protein products of three *male-specific lethal* genes (MSL-1, MSL-2, MSL-3), the *maleless* gene (MLE), the *males absent on the first* gene (MOF), and two long noncoding RNAs (*roX1* and *roX2*) (Samata and Akhtar 2018). The DCC initially binds to about 250 genomic regions, called chromosomal entry sites or high-affinity sites (HAS) (Alekseyenko et al. 2008; Straub et al. 2013). The HAS span the entire X chromosome, although most are located in gene-rich regions and at the boundaries of topologically associating domains (Alekseyenko et al. 2012; Ramírez et al. 2015). The location of HAS in regions with high connectivity allows the complex to spread over long distances to promote assembly of the DCC at other X-linked sites with lower affinity (Ramírez et al. 2015; Schauer et al. 2017). At each of the bound sites, the DCC directs acetylation of histone H4 on lysine 16 (H4Ac16), which is enriched at X-linked gene bodies and promotes elongation of RNA polymerase II. This results in an open chromatin structure and hypertranscription of genes in the exposed region (Ferrari et al. 2013; Kuroda et al. 2016).

In contrast to the soma, X-chromosome dosage compensation does not occur in the male germline (Meiklejohn et al. 2011; Meiklejohn and Presgraves 2012; Argyridou and Parsch 2018). Instead, the expression of X-linked genes is suppressed through a mechanism analogous to the meiotic sex chromosome inactivation that occurs in mammals (Lifschytz and Lindsley 1972; Hense et al. 2007; Vibranovski et al. 2009; Kemkemer et al. 2011, 2014; Meiklejohn et al. 2011). In *D. melanogaster*, studies have shown that X-linked reporter genes typically show three-to-six times lower expression in the male germline than their autosomal counterparts, with the degree of X suppression being dependent on the maximal expression level of the gene (Argyridou and Parsch 2018).

In addition to chromosome-wide differences in expression regulation between females and males, there are also many gene-specific differences in expression regulation between the sexes. A large proportion of *D. melanogaster* genes, both autosomal and X-linked, show differential expression between the sexes (Ellegren and Parsch 2007; Parsch and Ellegren 2013; Grath and Parsch 2016) and these sex-biased genes are not equally distributed among the X chromosome and the autosomes. The X chromosome is enriched for genes with female-biased expression (“feminization”) in whole flies and in various somatic tissues, including brains, heads, Malpighian tubules, and gonads (Huylmans and Parsch 2015). In contrast, a significant paucity of genes with male-biased expression (“demasculinization”) has been observed in studies of whole flies and gonads (Parisi et al. 2003; Ranz et al. 2003). This pattern of demasculinization does not hold for all tissues, however, as a significant overrepresentation of male-biased genes has been reported in studies of brain and head expression (Chang et al. 2011; Catalán et al. 2012; Huylmans and Parsch 2015; Khodursky et al. 2020). It has been hypothesized that the enrichment of sex-biased genes on the X chromosome in brain and head is related to dosage compensation, with genes located near DCC binding sites having greater upregulation of expression in males than genes located far from DCC binding sites (Huylmans and Parsch 2015). Consistent with this model, a negative correlation between a gene’s male-to-female expression ratio and its distance to the nearest DCC binding site has been observed for brain and head (Huylmans and Parsch 2015). In whole flies or gonads, which typically show a much greater degree of sex-biased expression, a positive correlation has been observed, suggesting that gene-specific regulation is the predominant driver of sex-biased expression and that DCC binding may interfere with sex-specific regulation (Bachtrog et al. 2010; Huylmans and Parsch 2015). Furthermore, it has been proposed that the head and brain may be more sensitive to dosage compensation than other tissues, as they show higher expression of the DCC components MSL-2 and MLE than other tissues (Straub et al. 2013; Huylmans and Parsch 2015; Vensko and Stone 2015).

Because previous studies of the effect of X-chromosomal location on gene expression were limited to endogenous genes, it was not possible to disentangle the influence of chromosomal location from that of gene-specific regulation. To overcome this limitation, we inserted an exogenous reporter gene at many unique locations across the X chromosome. The reporter gene consisted of the *Escherichia coli lacZ* gene under the control of a minimal human cytomegalovirus (CMV) promoter, which drives expression in multiple *D*. *melanogaster* tissues (Parsch 2004). By measuring reporter gene expression in both sexes and in different tissues, we could determine the effects of chromosomal context on gene expression, while avoiding the effects of gene-, tissue-, and sex-specific regulation that are common to endogenous genes. We found a negative correlation between a reporter gene’s male-to-female expression ratio and its distance to the nearest DCC binding site in somatic tissues but not in gonads, which is consistent with patterns seen for endogenous genes. The distance to a DCC binding site had a greater influence on reporter gene expression than local regulatory sequences affecting the native genes surrounding the insertion site. Although average levels of sex-biased expression did not differ between brain and head, there was greater positional effect variation in the brain, which may contribute to the relative excess of X-linked sex-biased genes observed in this tissue.

## Materials and Methods

### Reporter Gene Construct

The reporter gene construct contained two copies of the *Escherichia coli* β-galactosidase coding sequence (*lacZ*), each regulated by a CMV promoter (Parsch 2004). Two copies of the *lacZ* gene were included to provide a higher level of expression, particularly in relatively small tissue samples such as the gonad. The construct is flanked by the terminal repeat sequences of a *P* transposable element and includes the *D. melanogaster mini-white* gene as an eye color marker gene for easy identification of transformed flies.

### Mobilizing the Reporter Gene to New Chromosomal Locations

New X-linked insertions of the reporter gene were obtained by mobilization of the *P*-element vector as described in Hense et al. (2007). The mobilization scheme included several steps. First, females with an X-linked insertion of the reporter gene (marked by red eyes) were mated to *yw; Δ2-3, Sb/TM6* males containing a source of transposase on the third chromosome (marked by stubble bristles). Male offspring with red eyes and stubble bristles were mated to females of the *yw* background. If red-eyed males were obtained in the subsequent generation, this would indicate that the reporter gene moved from the X chromosome to an autosome. Cases in which red eyes and stubble bristles always appeared together would indicate a mobilization to the third chromosome containing the source of transposase. Males with this phenotype were used for further crosses with *yw* females to mobilize the transgene off of the third chromosome. In these cases, we collected offspring that had red eyes, but wild-type bristles. These flies carried new X-linked or autosomal insertions and were used for further mapping.

### Mapping Insertion Locations

To identify flies with a reporter gene insertion on the X chromosome, we crossed the males described above to *yw* females. If the insertion was on the X chromosome, all female offspring, but no male offspring, will inherit the red-eye marker. To find the exact chromosomal location of each reporter gene insertion, we used an inverse polymerase chain reaction (PCR) technique (Bellen et al. 2004). Five males and four females were homogenized and DNA was extracted using the MasterPure DNA Purification Kit (Epicentre, Madison, WI). Digestion and self-ligation were done using *Hin*PI or *Hpa*II restriction enzymes and T4 DNA ligase (New England Biolabs, Ipswich, MA). The DNA fragments containing the transgene and the unknown flanking sequence were PCR-amplified using primers specific to the transformation vector: Plac1–Plac4 (5′-CACCCAAGGCTCTGCTCCCACAAT-3′, 5′-ACTGTGCGT TAGGTCCTGTTCATTGTT-3′) and EY.3.F–EY.3.R (5′-CAATA AGTGCGAGTGAAAGG-3′, 5′-ACAATCATATCGCTGTCTCA C-3′). The subsequent sequencing of PCR products was performed using BigDye v1.1 chemistry on an ABI 3730 automated sequencer (Applied Biosystems, Foster City, CA) with two primers: Sp1 (5′-ACACAACCTTTCCTCTCAACAA-3′) and EY.3.F (above). The genomic locations of the insertions were determined by a BLAST search (Altschul et al. 1990) using the sequences flanking the transgene as the query and the *D. melanogaster* genome (Release 6.31) as the reference. A total of 102 transgenic lines with unique X-chromosomal insertions were obtained (supplementary file S1, Supplementary Material online). Some of these insertions were very close to each other. In order to avoid pseudoreplication in our analyses, we did not treat insertions within 500 bp of each other as independent replicates of different locations. Instead, they were treated as replicates of the same location. In total, there were 32 such insertions, which were present at 13 different genomic locations. After combining these 32 insertions as replicates of 13 locations, a total of 83 unique insertion locations remained. The coefficient of variation (CV) for reporter gene expression among replicates of the combined insertions was not greater than that among biological replicates of individual insertions (supplementary fig. S1, Supplementary Material online), indicating that short-range (within 500 bp) chromosomal effects on expression are negligible.

### Reporter Gene Expression Assays

The expression of the *lacZ* reporter gene was measured with a β-galactosidase enzymatic activity assay. Activity was measured in carcass (the whole fly with the gonads and head removed), head, testis, and ovary. Five hemizygous males or heterozygous females were used for protein extraction. In addition, homozygous females were assayed for a subset of 15 of the transgenic lines. For a subset of 32 transgenic lines, heads were dissected into brain and head case (the remaining head after brain extraction). Brains and head cases from ten hemizygous males and heterozygous females were used for protein extraction.

Each dissected tissue was homogenized in 200 µl (whole fly dissection) or 135 µl (head dissection) of lysis buffer (0.1 M Tris–HCl, 1 mM ethylenediaminetetraacetic acid, and 7 mM 2-mercaptoethanol; pH 7.5) to extract total protein. After 15 min of incubation of the homogenate on ice and centrifugation at 12,000 rpm and 4 °C, 50 µl of supernatant was taken from each sample for each of two technical replicates. Next, 50 µl of 2× assay buffer (200 mM sodium phosphate [pH 7.3], 2 mM MgCl_2_, 100 mM 2-mercaptoethanol, and 1.33 mg/ml *o*-nitrophenyl-β-d-galactopyranoside) was added to the protein extract and the change in absorbance was measured with a spectrophotometer for a total of 58 min at 420 nm at 37 °C. Enzyme activity was measured as the change in absorbance per minute (mOD/min) for the linear range of the reaction curve. For each transgenic line, as well as a *yw* negative control line, 2–4 biological replicates were tested, with the activity of each biological replicate corresponding to the mean of its two technical replicates.

To standardize enzymatic activity in samples derived from different tissues and sexes, the total soluble protein concentration was determined for each sample using the Lowry assay (Lowry et al. 1951). For each technical replicate, 10 µl (carcass, ovaries, and whole head) or 20 µl (brain, head case, and testis) of protein extract were diluted in 200 µl of water, followed by the addition of 200 µl of CTC working solution (0.025% (wt/vol) copper sulfate, 0.025% (wt/vol) potassium tartrate, and 2.5% (wt/vol) sodium carbonate, 0.2 N NaOH, 2.5% sodium dodecyl sulfate) and incubation for 10 min at room temperature. After adding 20% Folin and Ciocalteu’s phenol reagent (Sigma–Aldrich, Steinheim, Germany) and another incubation at room temperature for 30 min, the absorbance at 340 nm was measured for two technical replicates. Standardized activity (units/mg) was calculated as the enzyme activity divided by the amount of total protein in 1 ml. For subsequent analyses, standardized enzyme activity was used, unless otherwise indicated.

### Characterizing the Genomic Environment of Reporter Gene Insertions

The locations of DCC component binding sites (MLE, MSL2, and MSL3), as well as the HAS (defined by the colocalization of MLE and MSL2), were taken from previously published ChIP-chip (Alekseyenko et al. 2006) and ChIP-seq (Straub et al. 2013) studies. The distance between the binding sites of each DCC component and the reporter gene insertions was calculated as the minimum number base pairs between their starting (or ending) genomic coordinates. Because the regulatory effect of the DCC is thought to be limited to active chromatin compartments of ∼50 kb (Schauer et al. 2017), we limited our analysis to reporter genes located within this distance of a DCC binding site, which included the vast majority of the insertion locations (supplementary fig. S2, Supplementary Material online).

The location of each reporter gene insertion relative to the annotated genes of *D. melanogaster* was determined using FlyBase release 6.31 (Thurmond et al. 2019). We classified each insertion based on its position relative to the closest gene (5′ flanking or 3′ flanking for intergenic locations) or functional element of a gene (5′ UTR, coding sequence, intron, or 3′ UTR for intragenic locations). Eight of the insertions were in locations overlapping the transcriptional units of multiple genes. Four of these were cases in which one gene was embedded within a long intron of another gene. In these cases, we considered the insertion to be in the inner gene. The other four insertions were in locations where two genes had (partially) overlapping transcriptional units, depending on the mRNA isoform. In these cases, we considered the insertion to be in the gene that had its coding sequence closer to the insertion site.

To determine the sex bias of the endogenous genes in which the reporter genes were located, we used the log_2_(male/female) expression values compiled by Huylmans and Parsch (2015). This included expression data for brain (Catalán et al. 2012), head (Meisel et al. 2012), whole fly (Gnad and Parsch 2006), and gonads (Brown et al. 2014). Only genes with expression data in the above studies were included. For carcass, this amounted to 59 genes, whereas for both head and gonad it was 60 genes.

### Statistical Analysis

Correlation analyses were performed using both the Spearman rank correlation (*ρ*) and linear regression. For the main data set of 83 insertion locations, we compared β-galactosidase activity and log_2_(male/female β-galactosidase activity) in the different tissues by a paired *t*-test using the insertion locations as replicates. For smaller subsets of 15 and 32 transgenic lines (dosage and brain/head case analyses), we compared β-galactosidase activity and log_2_(male/female β-galactosidase activity) in the different sexes and tissues with a Wilcoxon signed-rank test using the independent transformed lines as replicates. The comparison of the groups of insertions with different proximity to the nearest DCC binding sites was carried out with an unpaired Wilcoxon signed-rank test. One-way analysis of covariance (ANCOVA) was performed for 64 insertions located within transcribed regions of genes to determine the effect of endogenous genes’ sex-biased expression (covariate) on log_2_(male/female β-galactosidase activity) in relation to the distance of each insertion to the nearest DCC binding site (independent variable). Two levels of distances were used: insertions within 25 kb (“close”) and insertions within the range of 25–50 kb (“distant”) to the DCC binding sites. One-way ANCOVA was performed using expression data from homozygous females, heterozygous females, and hemizygous males for 15 transgenic lines. For this, we first analyzed the influence of the distance to the nearest DCC binding site (covariate) on log_2_(male/female β-galactosidase activity) among homozygous and heterozygous females (independent variable). Second, we analyzed the influence of sex-biased expression of endogenous genes (covariate) on log_2_(male/female β-galactosidase activity) among these two groups of females (independent variable). To compare sources of variation among insertions in brain and head case, we used an *F*-test, a variance component analysis (Schützenmeister and Piepho 2012), and an asymptotic test for the equality of the CV (Feltz and Miller 1996).

## Results and Discussion

### Chromosomal Location and Expression of Reporter Genes

We obtained insertions of the *CMV–lacZ* reporter gene at 83 distinct X-chromosomal locations (supplementary fig. S3*A* and *B* and file S1, Supplementary Material online). All of the insertions were located in active chromatin regions and in proximity to genes, with 64 of them being located within transcribed regions (supplementary table S1, Supplementary Material online). This enrichment of reporter gene insertions in genic regions is consistent with the known insertion biases of the *P* transposable element included in our transformation vector (Bellen et al. 2004).

**Fig. 1:**
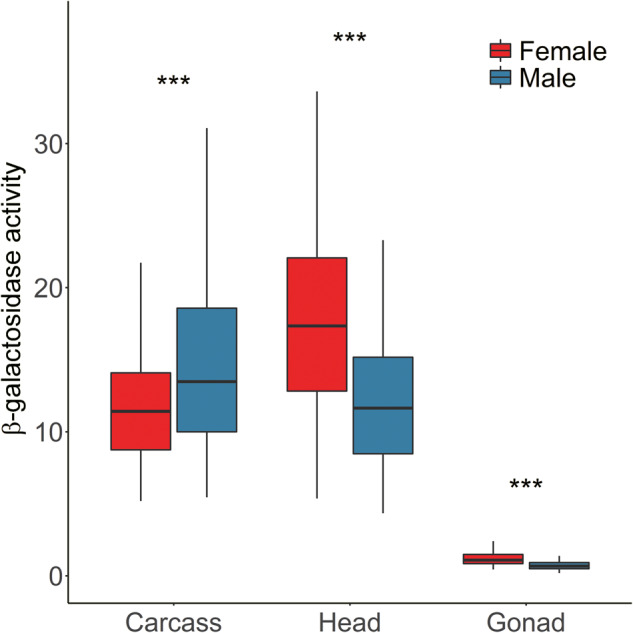
Reporter gene expression (measured as standardized β-galactosidase activity) in different sexes and tissues. For each tissue, differences between the sexes were tested using a paired *t*-test. ***P <* *0.001.

For each insertion location, reporter gene expression was measured in head, gonad, and carcass (here, defined as the whole fly with the head and gonads removed) using a β-galactosidase activity assay. To control for differences in X-chromosome gene dose between males and females, activity was measured in females that were heterozygous for the reporter gene insertion (i.e., both sexes had only one copy of the reporter construct). To account for potential differences in enzyme activity due to variation in body or tissue size, activity was standardized by the total amount of protein in the sample (supplementary file S1, Supplementary Material online). The total protein yield was higher in females than in males for all tissues, which reflects the larger body/gonad size of females. In carcass, the standardized β-galactosidase activity levels were significantly higher in males than in females (paired *t*-test, *P *=* *2.9 × 10^−7^) (fig. 1). This may be the result of partial dosage compensation of the X-linked reporter genes in males, which has been reported previously for somatic tissues (Argyridou and Parsch 2018). Because the reporter genes are present as a single copy in both sexes, X-chromosome dosage compensation is expected to lead to higher expression in males. Note, however, that the difference in expression between sexes is only about 1.2-fold, which is well below the 2-fold difference expected under complete dosage compensation. In the head, there was no evidence of the reporter genes being upregulated by X-chromosome dosage compensation. Instead, there was significantly higher expression in females than in males (paired *t*-test, *P *=* *1.2 × 10^−13^) (fig. 1). The reasons for this are unclear and such a pattern was not observed in a previous study that used a smaller number of reporter gene insertions (Argyridou and Parsch 2018). In gonads, the reporter genes also had significantly higher expression in females than in males (paired *t*-test, *P *<* *3.7 × 10^−13^) (fig. 1), which is consistent with the absence of dosage compensation in the male germline (Meiklejohn et al. 2011) and the general excess of female-biased gene expression observed in the ovaries (Huylmans and Parsch 2015). Although reporter gene activity was low in the testis relative to the other tissues, it was above that of negative controls (nontransgenic flies), for which the mean activity was zero (Argyridou and Parsch 2018). In addition, there were no significant differences in the level of variation of β-galactosidase activity between the testis and other tissues (asymptotic test for the equality of CV, *P *=* *0.139 for testis vs. head and *P *=* *0.094 for testis vs. carcass), indicating that reporter gene expression can be measured reliably in this tissue (supplementary fig. S1, Supplementary Material online).

### Sex-Biased Expression and the Influence of Endogenous Regulatory Elements

**Fig. 2:**
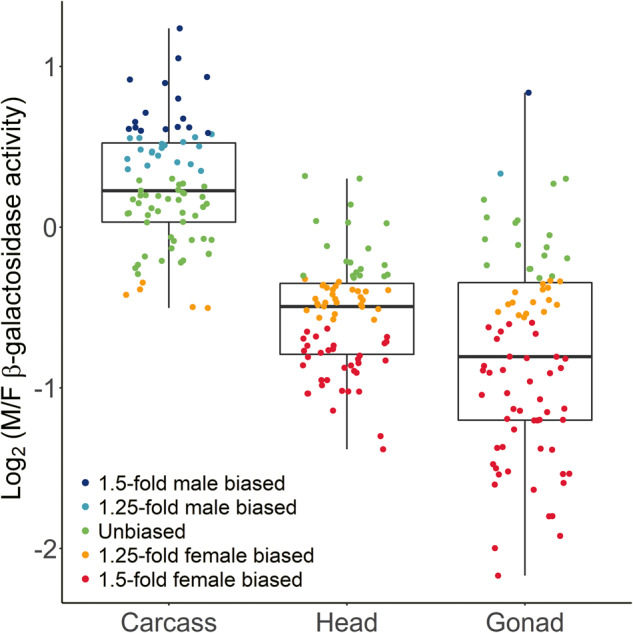
Male-to-female expression ratio (M/F) of reporter genes in different tissues. Colored points indicate the level of sex-biased expression for the individual insertion locations.

For each of the reporter gene insertions, we determined the degree of sex-biased expression by measuring the ratio of reporter gene activity between males and females (fig. 2). In general, the results were consistent with the levels of activity seen for the two sexes separately (fig. 1). In the carcass, there were more male-biased than female-biased genes (63 vs. 20). In contrast, for both the head and gonad, there was an excess of female-biased genes (6 vs. 77 in head, 8 vs. 75 in gonad). Interestingly, the male/female expression ratio of the insertions was positively correlated between carcass and head (*ρ* = 0.20, *P *=* *0.034), between carcass and gonad (*ρ* = 0.28, *P *=* *0.002), and between head and gonad (*ρ* = 0.15, *P *=* *0.103), which suggests that there are tissue-independent factors that influence the sex-biased expression of the reporter genes.

**Fig. 3:**
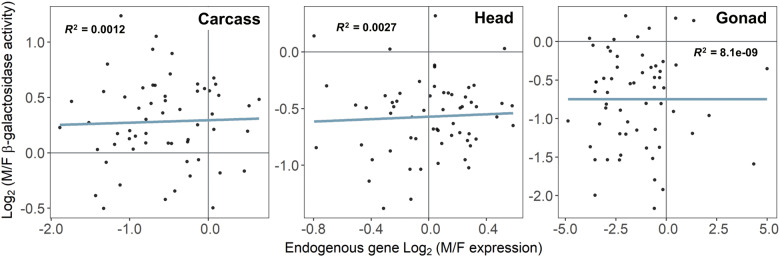
Correlation between the male-to-female expression ratio of each reporter gene and the endogenous gene in which it is located. Lines represent the least squares linear regression.

As mentioned above, 64 of the reporter gene insertions were located within the transcriptional units of genes (supplementary table S1, Supplementary Material online). Thus, it is possible that sequence elements regulating the expression of these genes may also influence the expression of the embedded reporter genes. To test this possibility, we compared the male/female expression ratio of the reporter genes with that of the endogenous genes in which they were located using previously published sex-biased expression data from the different tissues (Gnad and Parsch 2006; Catalán et al. 2012; Meisel et al. 2012; Brown et al. 2014; Huylmans and Parsch 2015). There was not a significant correlation between the sex-biased expression of the reporter genes and the endogenous genes in any of the tissues (Spearman rank correlation, *P *=* *0.062 in carcass, *P *=* *0.593 in head, and *P *=* *0.277 in gonad) (fig. 3). Thus, reporter gene expression shows little evidence of being influenced by local sex-specific regulatory elements associated with native *D. melanogaster* genes.

### The Effect of DCC Binding Site Proximity on Reporter Gene Expression

**Fig. 4:**
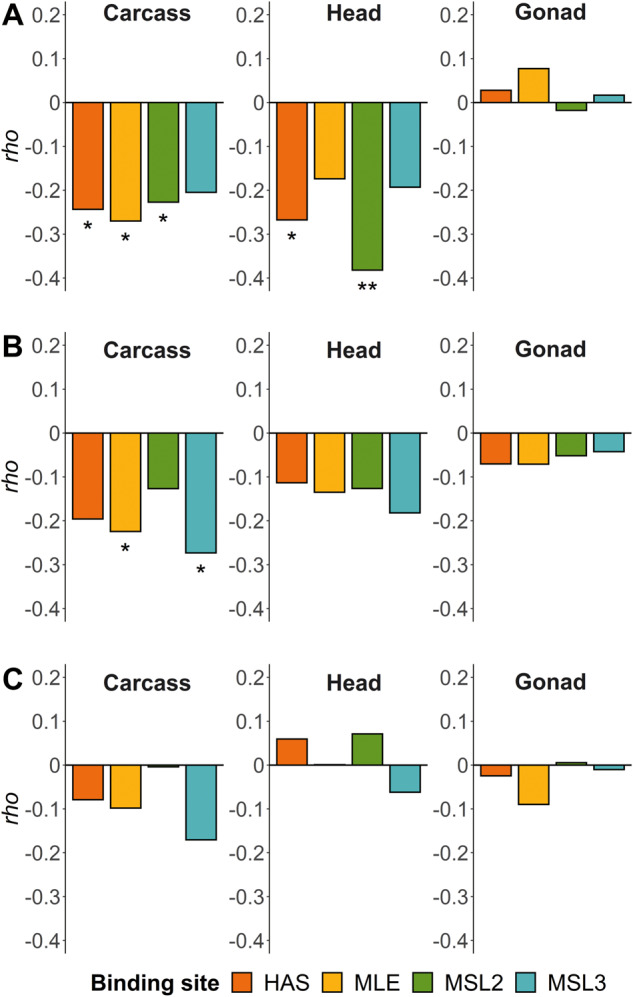
Spearman’s correlation coefficient (*ρ*) for the correlation between distance to the nearest DCC component binding sites and (*A*) male/female expression, (*B*) male expression, and (*C*) female expression. *P <* *0.05 and **P <* *0.01.

**Fig. 5:**
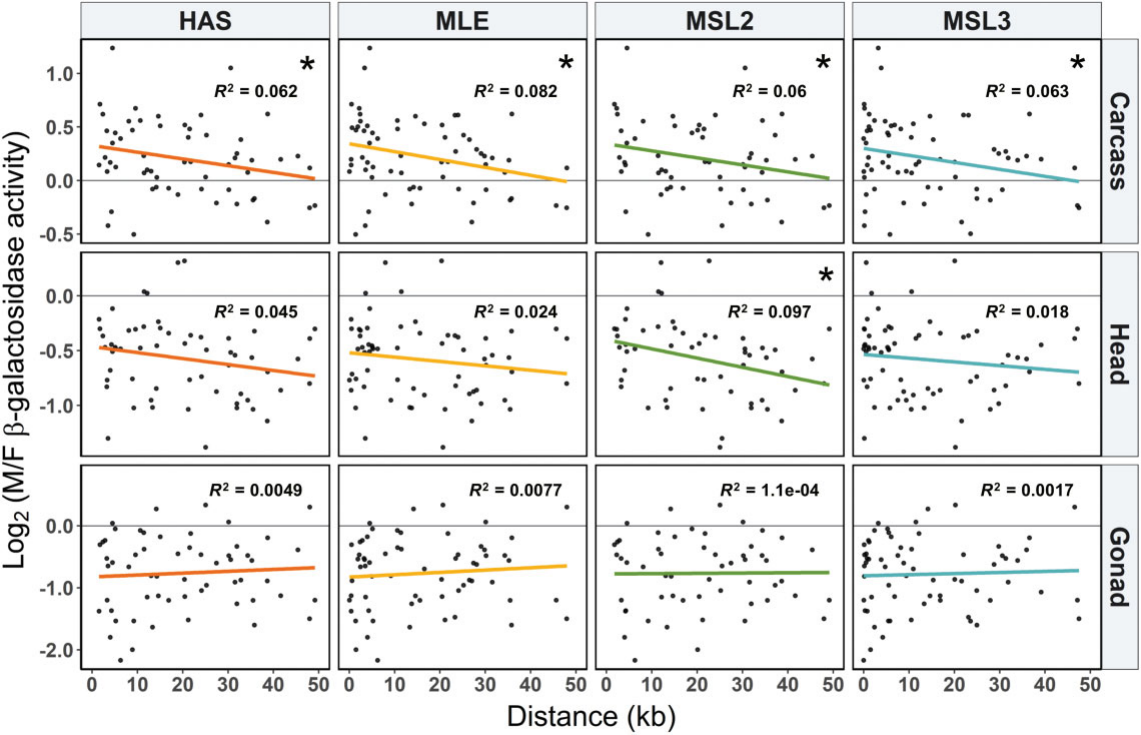
Male-to-female expression ratio (M/F) and distance to the nearest DCC component binding site (columns) for different tissues (rows). Colored lines represent the least squares linear regression. *P <* *0.05.

To test if the mechanism of dosage compensation influences the sex-biased expression of X-linked genes, we examined the correlation between the male-to-female expression ratio of each reporter gene insertion and its distance to the nearest DCC binding site (supplementary file S2, Supplementary Material online). For the somatic tissues (head and carcass), the correlation was consistently negative for all DCC components (figs. 4*A* and 5). This pattern was not observed for the gonad, where the correlation was close to zero and not significant (figs. 4*A* and 5). When only expression in males is considered, similar negative correlations are seen for all tissues (fig. 4*B* and supplementary fig. S4*A*, Supplementary Material online), which is consistent with dosage compensation affecting expression of the X chromosome in males. However, this negative correlation is weaker in the gonad, where dosage compensation is thought to be absent (although may occur in some of the somatic cells of the testis) (Meiklejohn et al. 2011; Meiklejohn and Presgraves 2012; Argyridou and Parsch 2018). In females, there is not a significant correlation between expression and distance to the binding site of any DCC component (fig. 4*C* and supplementary fig. S4*B*, Supplementary Material online), which is expected because the DCC does not assemble in females (Kelley et al. 1995). There is, however, a nonsignificant negative correlation between expression and binding-site distance for most DCC components in the female carcass, raising the possibility that these sites may also influence expression in females (Gallach and Betrán 2016).

A one-way ANCOVA was performed to examine the effect of the proximity of an insertion to the nearest DCC binding site on the male-to-female reporter gene expression ratio, after controlling for the sex-biased expression of the endogenous genes (supplementary file S2 and fig. S5, Supplementary Material online). In carcass, insertions located close to MLE and MSL2 binding sites had significantly higher male-to-female reporter gene expression (ANCOVA, *F *=* *3.9, df =1, 41, *P *=* *0.027 for MLE; *F *=* *3.2, df = 1, 37, *P *=* *0.042 for MSL2). In head, the same pattern was found for HAS (ANCOVA, *F *=* *3.6, df = 1, 39, *P *=* *0.032) and MSL2 (ANCOVA, *F *=* *7.3, df = 1, 38, *P *<* *0.01) binding sites.

### Reporter Gene Expression in Heterozygous and Homozygous Females

**Fig. 6:**
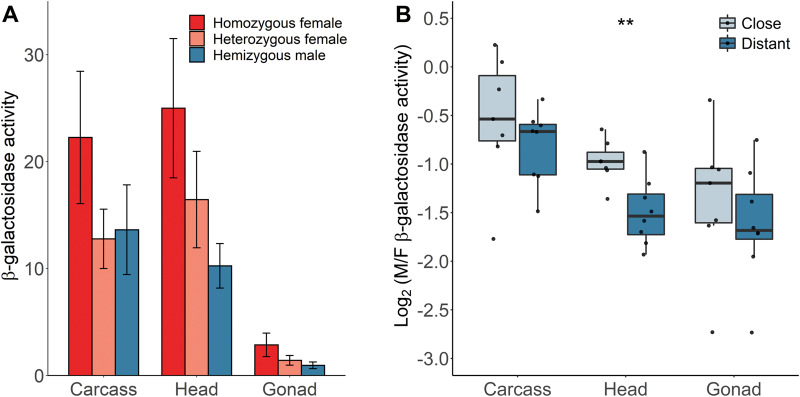
Effect of gene dose on β-galactosidase activity in various tissues. (*A*) Mean β-galactosidase activity for heterozygous females, homozygous females, and hemizygous males. Error bars indicate the standard deviation across insertions at different locations. (*B*) Male-to-female expression ratio (M/F) of reporter genes grouped by their proximity to the nearest HAS in different tissues for homozygous females and hemizygous males. Differences between the groups were tested with a Wilcoxon signed-rank test. **P <* *0.01.

Although our main analysis controlled for X-chromosome copy number differences between the sexes by comparing females heterozygous for the reporter gene insertion to hemizygous males, we also examined the effect of gene dose by measuring expression in homozygous females of 15 independent insertion lines. As expected, homozygous females had higher expression than heterozygous females in all tissues (fig. 6*A*). The ratio of homozygous to heterozygous female expression (95% CI) was 1.7 (1.45–2.08) in carcass, 1.5 (1.25–1.85) in head, and 2.0 (1.56–2.61) in ovary. In carcass and gonad, the 95% CI included 2, suggesting that there is a nearly linear relationship between expression and gene dose. In head, however, the expression ratio is below 2, suggesting that there is not a simple linear relationship between gene dose and expression in this tissue. The homozygous females also had higher expression than hemizygous males (fig. 6*A*), indicating that exogenous reporter genes introduced onto the X chromosome are not fully dosage compensated, which has been seen in previous studies (Laurie-Ahlberg and Stam 1987; Parsch et al. 1997; Argyridou and Parsch 2018). To test if there is some influence of dosage compensation on this subset of genes, we separated them into two distance categories on the basis of their proximity to each binding site, with seven (eight for MSL3) insertions being considered “close” (15–15,061 bp) and seven (MSL2 and MSL3) or eight (HAS and MLE) “distant” (24,987–47,502 bp). We then compared the expression ratios between hemizygous males and homozygous females. In all tissues and for all DCC binding sites, the “close” insertions showed higher relative expression in males than the “distant” insertions, although the difference between categories was only significant for the head (fig. 6*B* for HAS, supplementary fig. S6, Supplementary Material online, for MLE, MSL2, and MSL3).

For this subset of 15 reporter gene insertions, we tested how the dosage of the reporter gene in females, the expression pattern of the surrounding endogenous gene, and the proximity to the nearest DCC binding site affect the sex-biased expression of the reporter gene (supplementary file S2 and figs. S7 and S8, Supplementary Material online). In carcass, there was a significant positive correlation between the male-to-female expression ratio of the reporter genes and that of their surrounding endogenous genes (ANCOVA, *F *=* *4.7, df = 1, 20, *P *=* *0.043), and this pattern was similar for the groups with homozygous and heterozygous females (supplementary file S2 and fig. S7, Supplementary Material online). In all three tissues, after adjusting for the effect of endogenous genes, there was a significant difference in the reporter gene sex-biased expression between these two groups (ANCOVA, *F *=* *28.5, df = 1, 20, *P *<* *0.001 for the carcass; *F *=* *19.5, df = 1, 20, *P *<* *0.001 for the head; *F *=* *16.8, df = 1, 20, *P *<* *0.001 for the gonad). After adjusting for the effect of the distance to the nearest DCC binding site, there was also a significant difference in the reporter gene sex-biased expression between the groups of homozygous and heterozygous females in all tissues (supplementary file S2 and fig. S8, Supplementary Material online). In carcass, there was a significant effect of the distance to HAS, MLE, and MSL2 binding sites on sex-biased expression of the reporter gene. In head, this pattern was even stronger for all DCC binding sites (supplementary file S2 and fig. S8, Supplementary Material online). In gonad, we also found a negative correlation between the sex-biased expression of the reporter gene and the proximity to only HAS (ANCOVA, *F *=* *4.7, df = 1, 20, *P *=* *0.043).

### Reporter Gene Expression in the Brain and Head Case

**Fig. 7:**
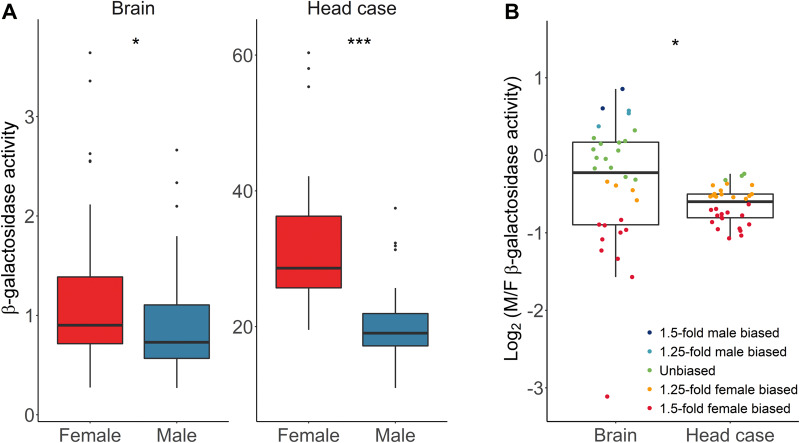
Reporter gene expression in brain and head case for (*A*) males and females, and (*B*) the ratio of male/female expression. Differences between sexes and tissues were tested with a paired Wilcoxon signed-rank test. *P <* *0.05 and ***P <* *0.001.

Previously it was found that, in the head, sex-biased genes were enriched on the X chromosome, and this enrichment was greater when considering only the brain (Huylmans and Parsch 2015). To test for expression differences between the brain and the rest of the head, we measured reporter gene expression in the brain and head case (the whole head with the brain removed) for a subset of 32 of our transgenic lines. Similar to the whole head (fig. 1), both tissues showed higher reporter gene activity in females (paired Wilcoxon signed-rank test, *P *=* *0.015 and *P *=* *4.7 × 10^−10^ for brain and head case, respectively), although the degree of female bias was higher in the head case than the brain (paired Wilcoxon signed-rank test, *P *=* *0.017) (fig. 7*A*).

**Fig. 8:**
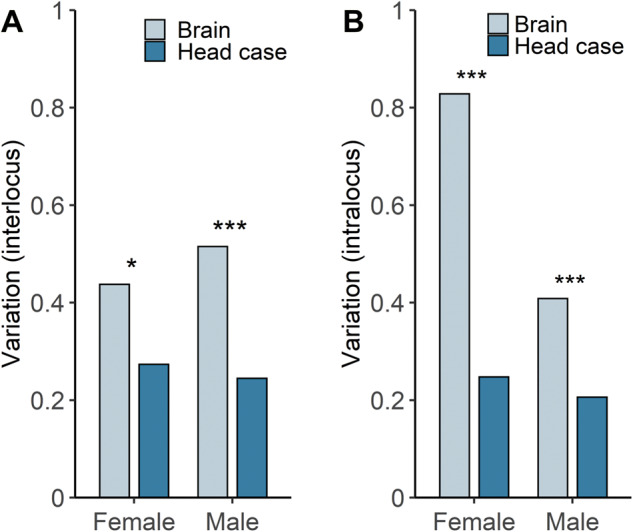
Sources of variation in β-galactosidase activity in brain and head case. (*A*) The interlocus component reflects variation among insertions at different X-chromosomal locations. (*B*) The intralocus component reflects variation among biological replicates of insertions at the same location. In all plots, variation is in units of standard deviation divided by the mean (CV). Differences between tissues were tested with an asymptotic test for the equality of CV. *P <* *0.05 and ***P <* *0.001.

When considering the male/female expression ratio of the individual reporter gene insertions, the median values were similar in brain and head case (fig. 7*B*). However, there was much greater variance among the insertion locations in the brain (*F*-test, *P *=* *5.6 × 10^−10^), with some showing strong male- or female-biased expression (fig. 7*B*). The difference in variance between the tissues could have two causes. First, it could be that there is greater technical variation among repeated activity measurements in the brain (intralocus variation), possibly because it is a smaller tissue with relatively low levels of reporter gene expression. Second, it could be that there is greater position-effect variation (interlocus variation) among the X-chromosomal insertion locations in the brain. To determine the contributions of these two factors to the overall variance, we carried out a variance component analysis (Schützenmeister and Piepho 2012) (fig. 8*A* and *B*). Although the brain displayed higher intralocus variation than the head case in both sexes (asymptotic test for the equality of CV, *P *=* *0.021 and *P *=* *4.8 × 10^−4^ for females and males, respectively), it also displayed significantly higher interlocus variation (asymptotic test for the equality of CV, *P *=* *1.7 × 10^−6^ and *P *=* *7.8 × 10^−4^ for females and males, respectively). Thus, gene expression appears to be more sensitive to chromosomal location in the brain than in the rest of the head.

## Conclusions

By using an exogenous reporter gene inserted at many X-chromosomal locations, we were able to test hypotheses about expression regulation without the confounding factor of gene-specific regulation that is common to studies of native genes. For example, we could directly test whether genes located near DCC binding sites showed higher expression in males (and a higher male/female expression ratio) than those located far away. Our results confirm this prediction for somatic tissues, where X-chromosome dosage compensation occurs in males. We did not see this effect in the gonad, where X-chromosome dosage compensation is absent. Although previous studies of endogenous gene expression reported a positive correlation between male-biased expression and the distance to the nearest DCC binding site in gonad, this was thought to be the result of selection to prevent interference between the dosage compensation machinery and the gene-specific regulation of testis-biased genes, which typically display strong tissue-specific regulation (Bachtrog et al. 2010; Huylmans and Parsch 2015). Our results are consistent with this interpretation, as our reporter genes should not be affected by the sex- or tissue-specific regulation that affects the native *D. melanogaster* genes. Because the gonads are enriched with sex-biased genes, especially those with a very high degree of sex bias, one might expect that the expression patterns observed in studies of whole flies would be more similar to those seen in gonad than in somatic tissues. Indeed, this is what has been reported for whole-fly expression data (Huylmans and Parsch 2015). In a previous study of gonadectomized flies (Parisi et al. 2003), a positive correlation between male-biased expression and the distance to the nearest DCC binding site was observed (Huylmans and Parsch 2015). That is, the relationship between DCC distance and male-biased expression was more similar to that seen in gonad than in somatic tissues. However, these expression data were from an early microarray study that detected only a few hundred sex-biased genes (Parisi et al. 2003), which may limit statistical power. Furthermore, the previous gonadectomized sample appears to have included residual gonadal transcripts, as the expression of several testis-specific genes was detected (Vensko and Stone 2014). For these reasons, we believe that the proximity to a DCC binding site has a similar affect in all somatic tissues, which is consistent with the pattern we see for head and carcass. The head and/or brain do not appear to be unusual in this regard. The brain, however, does appear to be unusual in that it shows a strong enrichment of sex-biased genes, particularly male-biased genes, on the X chromosome compared with other tissues. Our results suggest that this may be explained, at least partly, by an increased level of expression variation among regions of the X chromosome, which we observe both sexes. Because sex-biased expression is calculated as the ratio of the expression measured independently in each sex, the variation will be amplified in the male/female ratio, leading to greater variation and more cases of sex-biased expression.

We expect that the effects of dosage compensation on sex-biased gene expression reported here should be present only in taxa in which there is upregulation of the hemizygous sex chromosome in the heterogametic sex. In mammals, where sex chromosome dosage compensation occurs through inactivation of one of the X chromosomes in females, it is more likely that sex-biased expression will be influenced by variation in inactivation across the X chromosome, leading to female-biased expression of genes in regions that escape X-inactivation (Carrel and Willard 2005). In some female-heterogametic taxa, such as birds, sex-biased expression is more likely to be influenced by an absence of dosage compensation in females, leading to widespread male-biased expression of Z-linked genes (Ellegren et al. 2007; Itoh et al. 2007). It has recently been reported that a female-heterogametic Lepidopteran with a neo-Z chromosome (Monarch butterfly) displays two distinct modes of sex chromosome dosage compensation (Gu et al. 2019): the ancestral Z is downregulated in ZZ males in a manner similar to that seen for the X chromosome in *Caenorhabditis elegans* females, whereas the neo-Z is upregulated in ZW females in a manner similar to that seen for the X chromosome in *D. melanogaster* males. The molecular mechanism responsible for neo-Z chromosome upregulation in Monarch females is not fully understood but, similar to *Drosophila*, it is associated with H4Ac16 (Gu et al. 2019). It is currently not known whether the degree of this upregulation varies with Z-chromosomal location in a way analogous to that seen in *D. melanogaster* males.

## Supplementary Material

Supplementary data are available at *Genome Biology and Evolution* online.

## Supplementary Material

evaa227_Supplementary_Data
