# Supplementary material for: The Influence of Chromosomal Environment on X-Linked Gene Expression in Drosophila melanogaster
Source: Genome Biol Evol. 2020 Oct 26;12(12):2391–402. doi: 10.1093/gbe/evaa227 (PMC7719225; doi:10.1093/gbe/evaa227)
Supplement: evaa227_Supplementary_Data [file evaa227_supplementary_data.zip › SuppFigsTabs.pdf]

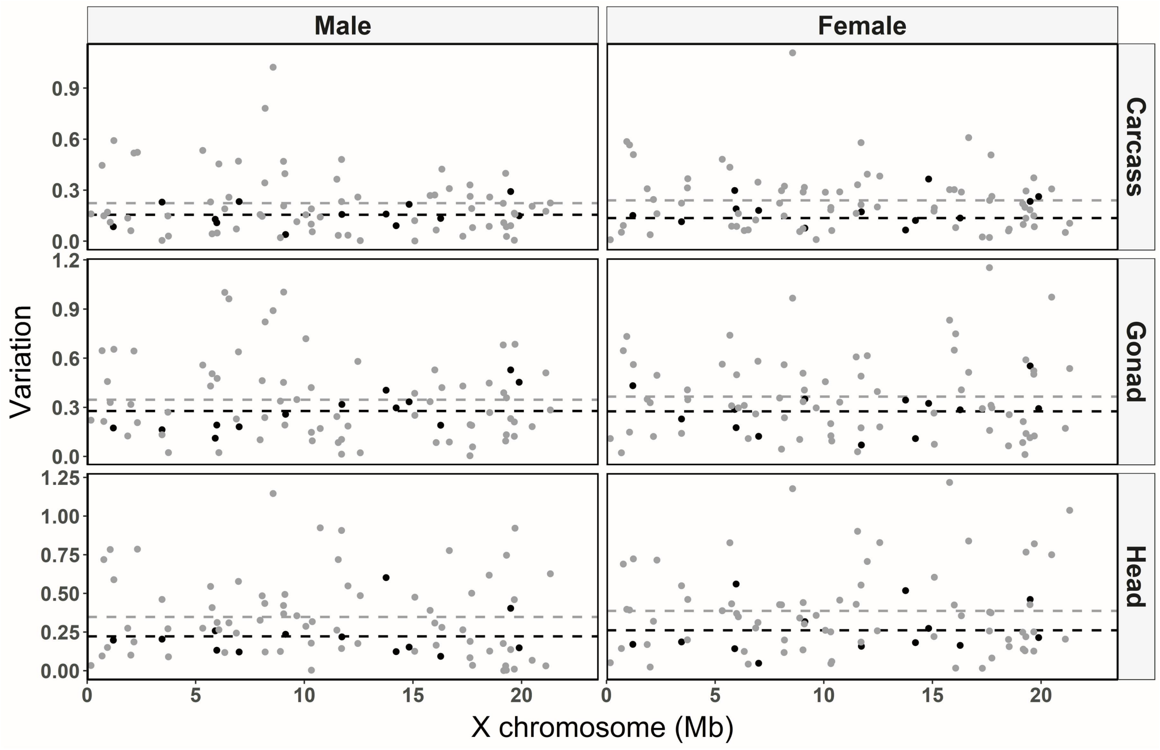

Fig. S1. Variation of β-galactosidase activity among biological replicates for 13 combined insertions (black dots) and 70 unique insertions (gray dots) in different sexes and tissues. Dashed lines indicate the mean level of variation for 13 combined insertions (black) and 70 unique insertions (gray). In all plots, variation is in units of standard deviation divided by the mean.

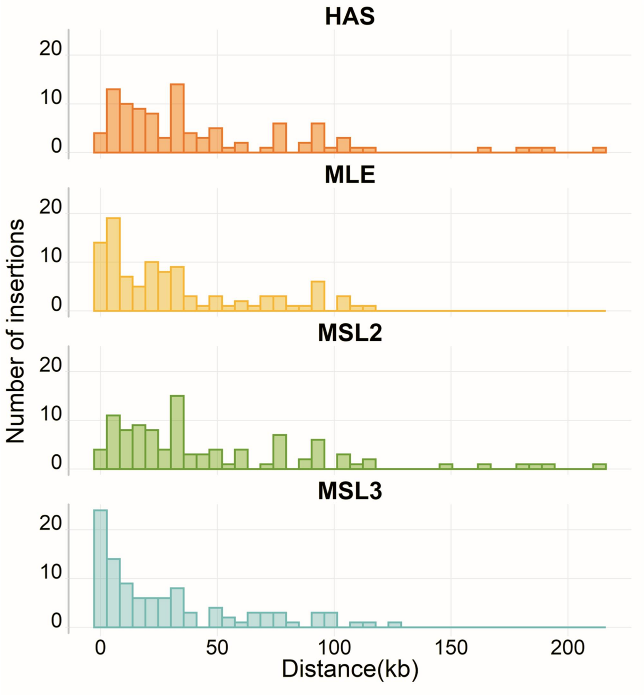

Fig. S2. Histograms of the minimum distance between reporter gene insertion sites and the binding sites of different DCC components.

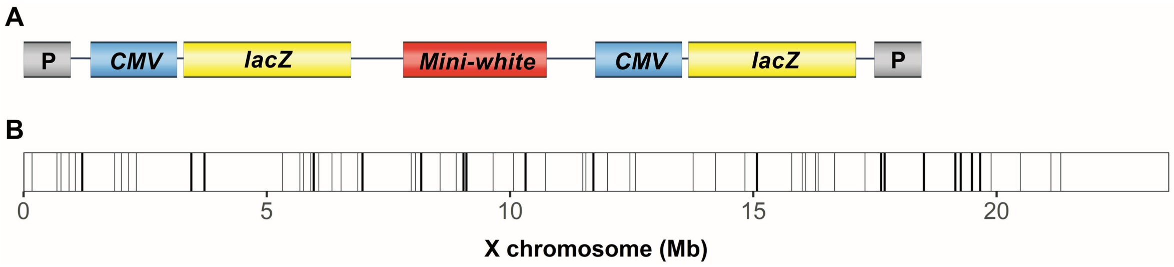

Fig. S3. Reporter gene design and insertion locations along the X chromosome. (*A*) Schematic diagram of the *CMV-lacZ* reporter construct, which contains two copies of the *E. coli lacZ* gene fused to a minimal human cytomegalovirus (CMV) promoter flanked by the terminal repeat sequences of a *P* transposable element. The *D. melanogaster mini-white* gene is included as a visible eye color marker gene. (*B*) Insertion locations on the X chromosome. Vertical lines show the locations of the 83 reporter gene insertions. Bold lines indicate locations where two insertions are within 50 kb of each other.

Table S1. Number of reporter gene insertions in different gene regions.

Gene region	Insertions
5' flanking	17
5' UTR	28 (7)[a]
Coding	1
Intron	30
3' UTR	5 (1)[a]
3' flanking	2

[a]Numbers in parentheses indicate insertions that could also be classified as intronic, depending on the transcript isoform.

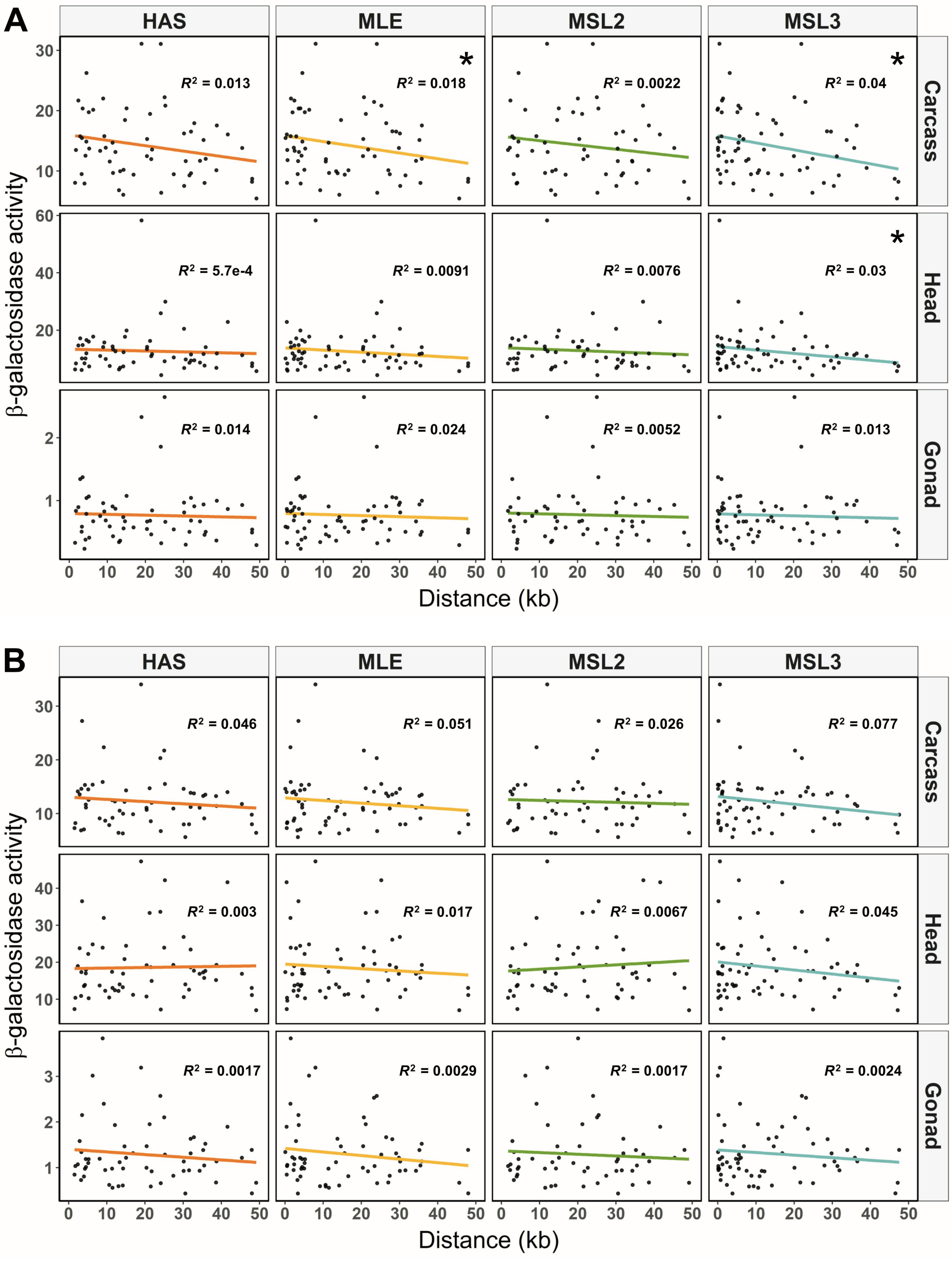

Fig. S4. Reporter gene expression (measured as standardized β-galactosidase activity) and distance to the nearest DCC binding site (columns) for different tissues (rows) in (*A*) males and (*B*) females. Colored lines represent the least squares linear regression. Dots represent the individual insertions. **P* < 0.05.

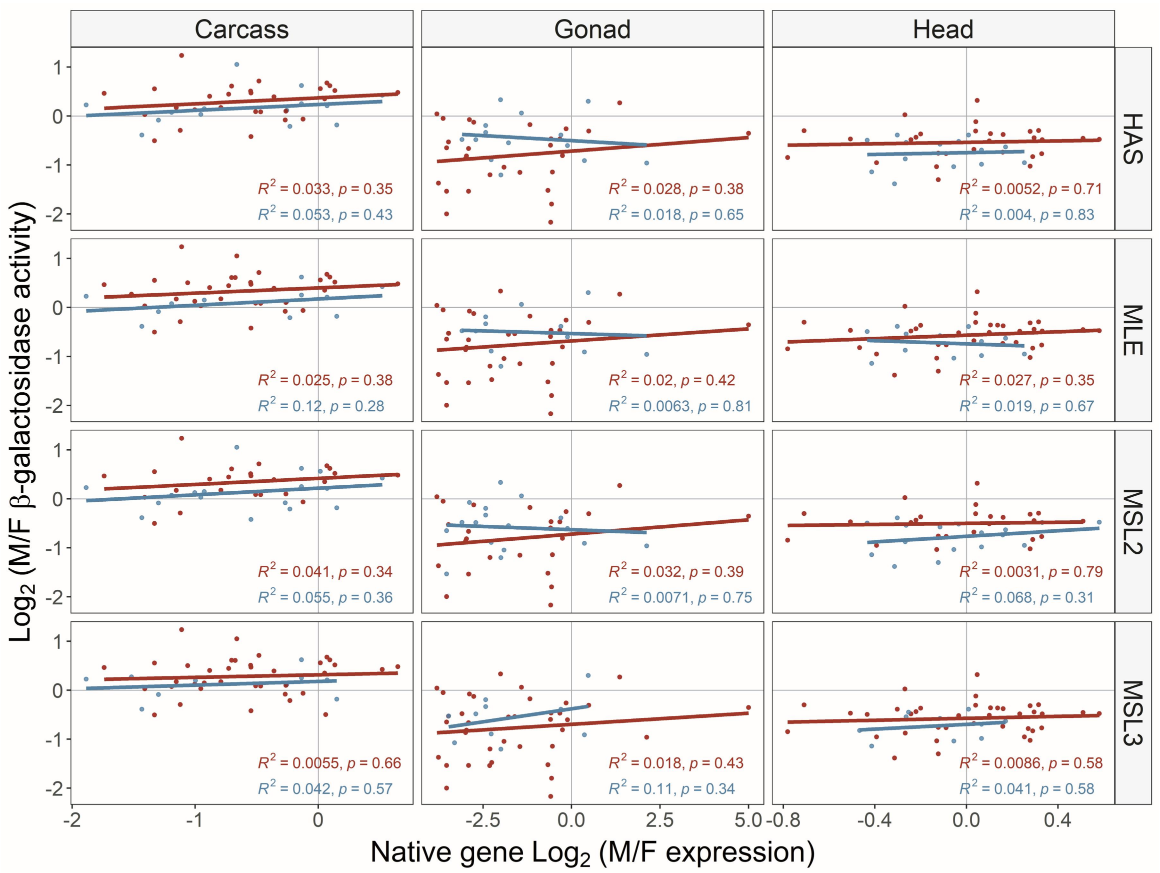

Fig. S5. Male-to-female expression ratio of each reporter gene and the endogenous gene in which it is located for groups of their proximity to the nearest DCC binding site (rows) in different tissues (columns). Lines represent the least squares linear regression. Dots represent the individual insertions. Color indicates 'close' (red) and 'distant' (blue) groups of close proximity to the inserts.

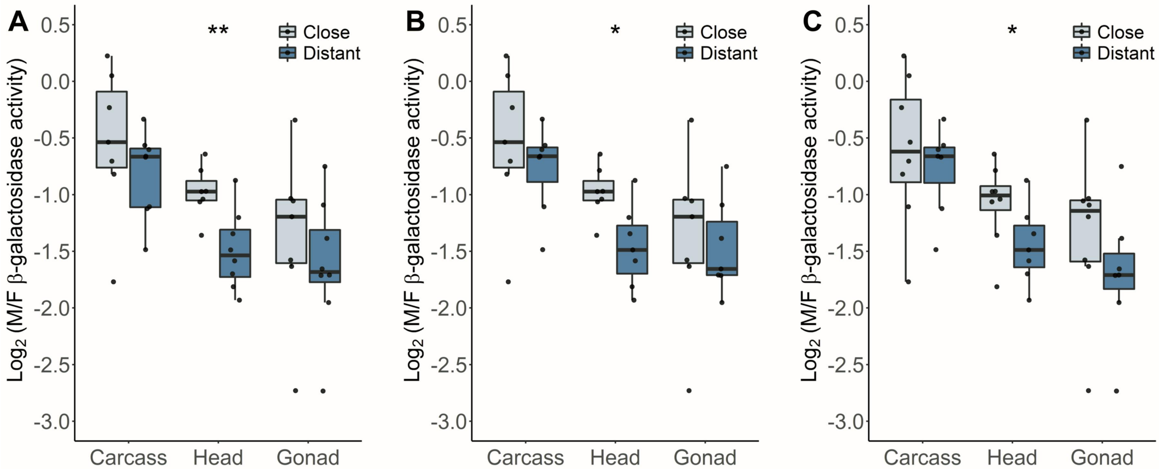

Fig. S6. Male-to-female expression ratio (M/F) of reporter genes grouped by their proximity to the nearest (*A*) MLE, (*B*) MSL2, or (*C*) MSL3 binding site in different tissues for homozygous females and hemizygous males. Differences between tissues were tested with a Wilcoxon signed-rank test. $*P < 0.05$, $**P < 0.01$.

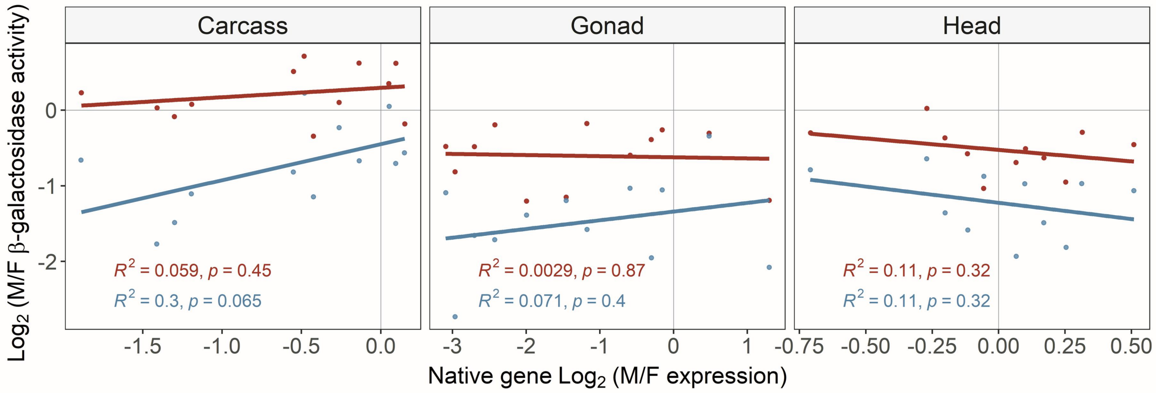

Fig. S7. Male-to-female expression ratio of each reporter gene and the endogenous gene in which it is located for the groups of the dosages of the reporter gene (heterozygous and homozygous females) in different tissues. Lines represent the least squares linear regression. Dots represent the individual insertions. Color indicates the source of data on the reporter gene sex-biased expression; heterozygous females and hemizygous males (red); homozygous females and hemizygous males (blue).

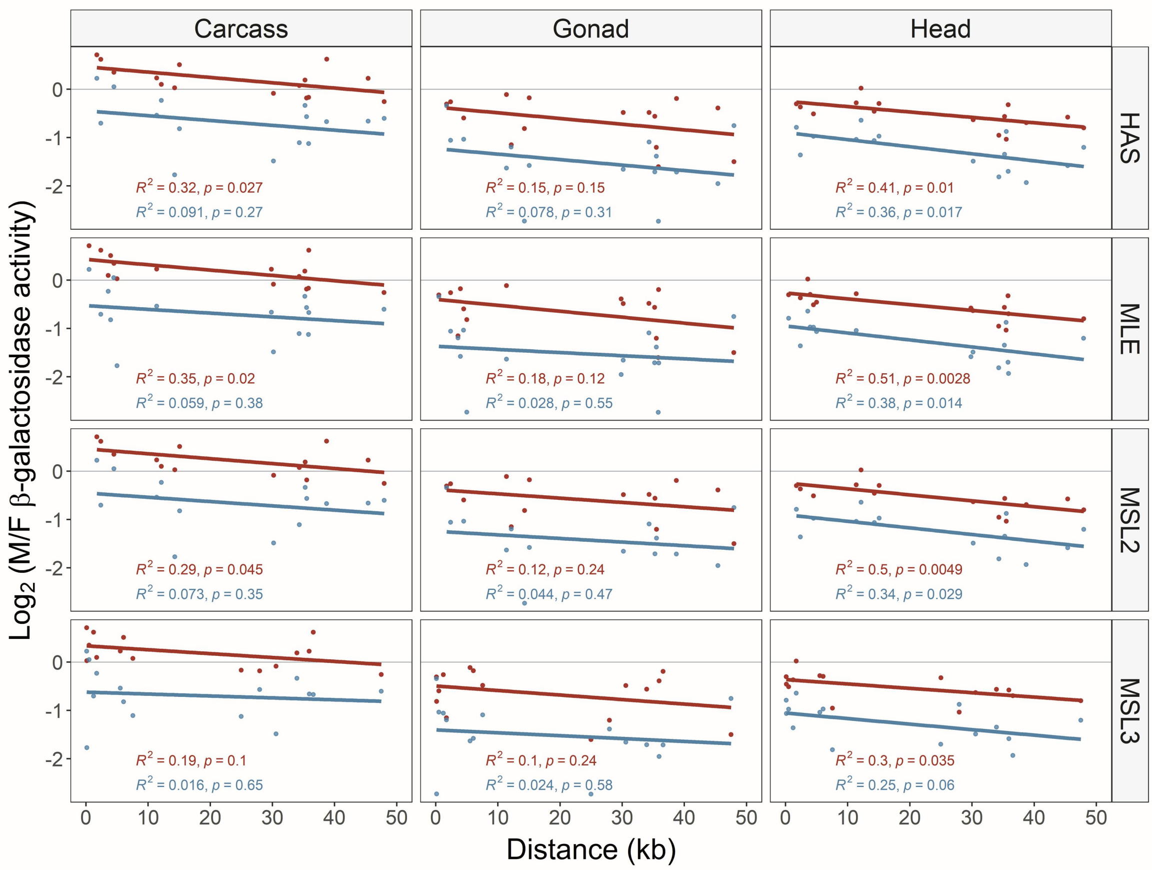

Fig. S8. Male-to-female expression ratio of each reporter gene and distance to the nearest DCC component binding site (rows) for the groups of the dosages of the reporter gene (heterozygous and homozygous females) in different tissues (columns). Lines represent the least squares linear regression. Dots represent the individual insertions. Color indicates the source of data on the reporter gene sex-biased expression; heterozygous females and hemizygous males (red); homozygous females and hemizygous males (blue).
